# The do’s and dont’s of liver CT and MR imaging

**DOI:** 10.1186/1470-7330-14-S1-O12

**Published:** 2014-10-09

**Authors:** Wolfgang Schima

**Affiliations:** 1 Department of Diagnostic and Interventional Radiology, Krankenhaus Goettlicher Heiland, KH der Barmherzigen Schwestern, and Sankt Josef-Krankenhaus, Vinzenzgruppe, Dornbacher Strasse 20-28, 1170 Vienna, Austria

## 

Contrast enhanced multi-detector CT (MD CT) is now the most commonly used imaging modality for detection of liver metastases and work-up of equivocal lesions found at ultrasound. MR imaging is an established technique for non-invasive characterisation of liver masses and for preoperative potentially resectable liver tumours. Diagnostic value of each modality strongly depends on the scan technique. With MDCT, the role of thin slice imaging has been well established, with slice thicknesses of 5–7.5 mm being inferior to thinner slices in terms of lesion detection [1]. The amount of contrast material as well as the flow rate influence enhancement of hypervascular lesions in the arterial phase and the magnitude of liver parenchyma enhancement in the venous phase, respectively [2,3]. The number of CT scans varies with the local indication, with single-phasic venous scans to 4-phasic scans, as recommended by European and US guidelines for HCC detection and characterisation.

For MR imaging, a multi-point DIXON technique has replaced conventional T1-weighted GRE pulse sequences; with this technique in one breathhold in-phase, opposed-phase, fat-suppressed, and water-suppressed images can be obtained, without any spatial misregistration. Diffusion-weighted pulse sequences significantly improve detection of metastases [4,5]. As a black-blood technique with T2-weighted image impression, it demonstrates small lesions without any blurring by an adjacent high signal intensity of vessels of bile ducts [6]. Administration of contrast agents is mandatory for liver MRI, with the choice of either non-specific gadolinium chelates or liver specific contrast agents. Indications for liver-specific contrast agents include preoperative evaluation of liver metastases and characterisation of hepatocellular lesions (FNH, adenoma), whereas non-specific gadolinium chelates are used for characterisation of haemangiomas, after liver resection or tumour ablation. The choice of the imaging techniques and the scan or contrast agent protocol should be tailored according to the clinical question.

## References

[B1] Weg N Scheer MR Gabor MP Liver lesions: improved detection with dual-detector-array CT and routine 2.5-mm thin collimation Radiology 1998 209 417 426 10.1148/radiology.209.2.9807568 9807568

[B2] Han JK Choi BI Kim AY Kim SJ Contrast media in abdominal computed tomography: optimization of delivery methods Korean J Radiol 2001 2 28 36 10.3348/kjr.2001.2.1.28 11752966 PMC2718092

[B3] Johnson PT Fishman EK IV contrast selection for MDCT: current thoughts and practice AJR Am J Roentgenol 2006 186 406 415 10.2214/AJR.04.1902 16423946

[B4] Holzapfel K Reiser-Erkan C Fingerle AA Comparison of diffusion-weighted MR imaging and multidetector-row CT in the detection of liver metastases in patients operated for pancreatic cancer Abdom Imaging 2011 36 179 184 10.1007/s00261-010-9633-5 20563868

[B5] Koh DM Brown G Riddell AM Detection of colorectal hepatic metastases using MnDPDP MR imaging and diffusion-weighted imaging (DWI)alone and in combination Eur Radiol 2008 18 903 910 10.1007/s00330-007-0847-z 18193234

[B6] Parikh T Drew SJ Lee VS Focal liver lesion detection and characterization with diffusion-weighted MR imaging: comparison with standard breath-hold T2-weighted imaging Radiology 2008 246 812 822 10.1148/radiol.2463070432 18223123

